# Supplementary material for: Transcriptome Analysis Reveals Vimentin-Induced Disruption of Cell–Cell Associations Augments Breast Cancer Cell Migration
Source: Cells. 2022 Dec 13;11(24):4035. doi: 10.3390/cells11244035 (PMC9776984; doi:10.3390/cells11244035)
Supplement: Supplementary file 1 [file cells-11-04035-s001.zip › DEGs and enrichment analysis.zip!/DEGs and enrichment analysis/WT_VIMvsVector KEGG/UP/WT_VIMvsVector_UP.pathway_map.zip!/WT_VIMvsVector_UP.pathway_map/src/hsa04810.html]

hsa04810


- hsa:5216

- Up regulated genes

ENSG00000233328(2.59819417783962)

- hsa:5216

- Up regulated genes

ENSG00000233328(2.59819417783962)

- hsa:1128

- Up regulated genes

ENSG00000168539(2.56985566842982)

- hsa:3674

- Up regulated genes

ENSG00000005961(3.91653577454454)

- hsa:2264

- Up regulated genes

ENSG00000160867(2.86168022046666)

Close
